# The minimal informative monitoring interval of N-terminal pro-B-type natriuretic peptide in patients with stable heart failure

**DOI:** 10.1186/s12872-020-01537-7

**Published:** 2020-06-01

**Authors:** Zhehao Dai, Taku Asano, Osamu Takahashi, Nobuyuki Komiyama, Sachiko Ohde

**Affiliations:** 1 Department of Cardiology, St. Luke’s International Hospital, 9-1 Akashi-cho, Chuo-ku, Tokyo, 104-0045 Japan; 2 Graduate School of Public Health, St. Luke’s International University, 3-6-2 Tsukiji, Chuo-ku, Tokyo, 104-0045 Japan

**Keywords:** N-terminal pro-B-type natriuretic peptide (NT-proBNP), Heart failure, Minimal informative monitoring interval, Signal-to-noise ratio

## Abstract

**Background:**

N-terminal pro-B-type natriuretic peptide (NT-proBNP) is a potential biomarker for monitoring the status of heart failure. However, the optimal monitoring interval of NT-proBNP is unknown. This study sought to investigate the minimal informative monitoring interval of NT-proBNP in patients with stable chronic heart failure.

**Methods:**

This retrospective cohort study included patients who were admitted due to heart failure and subsequently followed with serial NT-proBNP measurements in a tertiary hospital. We analyzed NT-proBNP measured between six months after discharge and the earliest timepoint of: an alteration of medication regimen, readmission due to worsening of heart failure, or all-cause death. To distinguish progression of the disease from biological variability and measurement error, the signal-to-noise ratio method was applied with a random-effects model.

**Results:**

In the 368 patients included, NT-proBNP was measured for a median 6 times. In the random-effects model, signal (progression of disease) exceeded noise (biological variability and measurement error) at 7.9 months (95% confidence interval [CI]: 5.1–9.6), while noise corresponded to a 61% increase from baseline. In stratified analysis using the AHEAD risk score, the minimal informative monitoring interval shortened as the risk score increased (0–1 point: 12.2 months [95%CI: 10.3–14.4]; 2–3 points: 8.0 months [95%CI: 6.8–9.7]; 4–5 points: 3.3 months [95%CI: 3.0–3.8]).

**Conclusions:**

In patients with stable chronic heart failure, the minimal informative monitoring interval of NT-proBNP measurement was 7.9 months in the current population, which varied with underlying risks. The optimal monitoring interval could be lengthened for patients at lower risks.

## Background

Biochemical markers, including natriuretic peptides such as N-terminal pro-B-type natriuretic peptide (NT-proBNP), are widely used in the clinical management of heart failure. The serum level of NT-proBNP can reliably be used as a diagnostic tool and a prognostic predictor throughout the wide spectrum of acute and chronic heart failure [1–5]. Moreover, recent studies reported potential utility of NT-proBNP for monitoring chronic heart failure and titrating medical therapy [6–8]. However, this still remains controversial [9–12], and no established consensus has been reached regarding the monitoring interval of NT-proBNP in patients with chronic heart failure.

Several studies have reported that serum NT-proBNP was measured routinely every 3 months upon follow-up visits in clinical management of chronic heart failure [9, 12]. Nevertheless, NT-proBNP measurement is susceptible to up to 40% of intra-individual variability without disease progression, which hinders the detection of a true change in the disease status [13–16]. Frequent measurements, therefore, may lead to therapeutic confusion as well as unnecessary costs. Thus, it is crucial to clarify the minimal informative interval for monitoring NT-proBNP in these patients. The purpose of the present study is to investigate the minimal informative interval for testing serum NT-proBNP level in patients with stable heart failure using real-world data, taking the intra-individual variability into account.

## Methods

### Study design

A single-center retrospective open-cohort study was conducted to identify the true progression and intra-individual variability, as well as to investigate a minimal informative interval of NT-proBNP measurement in patients with stable chronic heart failure. The present study was approved by the institutional research board of St. Luke’s International University (NO. 18-R070). All patients provided informed consent under an opt-out policy.

### Study population

Consecutive adult patients (aged 20 or more) who had been hospitalized at St. Luke’s International Hospital (Tokyo, Japan) with a diagnosis of heart failure from January 2003 to December 2017 were assessed for enrollment in the present study. The diagnosis of heart failure was based on Framingham criteria [17]. The following patients were excluded: patients 1) who had readmissions for heart failure within 6 months after the index admission; 2) with less than 3 times of NT-proBNP measurement during the analytic period defined below; 3) without any record of left ventricular ejection fraction assessed on transthoracic echocardiography at the time of index admission; 4) with end-stage renal disease requiring either hemodialysis or peritoneal dialysis; 5) who declined the consent. Those patients who had unplanned visits within 6 months after the index admission but were not readmitted, were not excluded. For patients who had multiple admissions due to heart failure during the study period, the first was registered as the index admission.

### Definition of analytic period in the present study

We excluded the NT-proBNP measurements acquired in the first 6 months after the index admission in order to allow a window period for stabilization of both heart failure and NT-proBNP [18]. We defined the first measurement of NT-proBNP after 6 months of the index admission as the baseline (i.e. the start of the analytic period). The endpoint of the analytic period was defined as the earliest timepoint at which either a physician changed the regimen of medication for heart failure, when the patient was readmitted for heart failure, or when the patient died from any cause **(Online Supplemental Fig.** **1****)**. The medication for heart failure included beta-blockers, angiotensin converting enzyme inhibitors, angiotensin receptor blockers, diuretics (thiazides, loop diuretics, mineralocorticoid receptor antagonists, and vasopressin V2-receptor antagonists), digoxin, and pimobendan. As this was a retrospective study, the schedules of outpatient visits and NT-proBNP measurements were left to each physician’s discretion.

### Data collection

Clinical and demographic data were collected from the patients’ electronic medical records. They included demographic data (gender, age, body weight, and height), comorbidities (hypertension, diabetes mellitus, dyslipidemia, ischemic heart disease, valvular disease, and kidney disease), smoking status, laboratory data, echocardiographic data, and prescriptions at each outpatient visit. Analyzed laboratory tests were all measured within the study site, and serum NT-proBNP was measured with an ECLusys NT-proBNP II kit (Roche Diagnostics Japan, Inc., Tokyo, Japan). All data were collected from electronic medical records by 2 healthcare information technicians and an independent physician who were not involved in the treatment and care of any of the participating patients.

### Statistical analysis

Continuous variables are presented as the median (interquartile range [IQR]) or the mean (standard deviation [SD]) as appropriate. Categorical variables are presented as number (percentage). Statistical analysis and model fitting were performed and visualized using R 3.5.2 with the R packages of “dplyr”, “lme4”, “performance”, “ggplot2”, and “forestplot” [19].

#### Random-effects model and signal-to-noise ratio

**Fig. 1:**
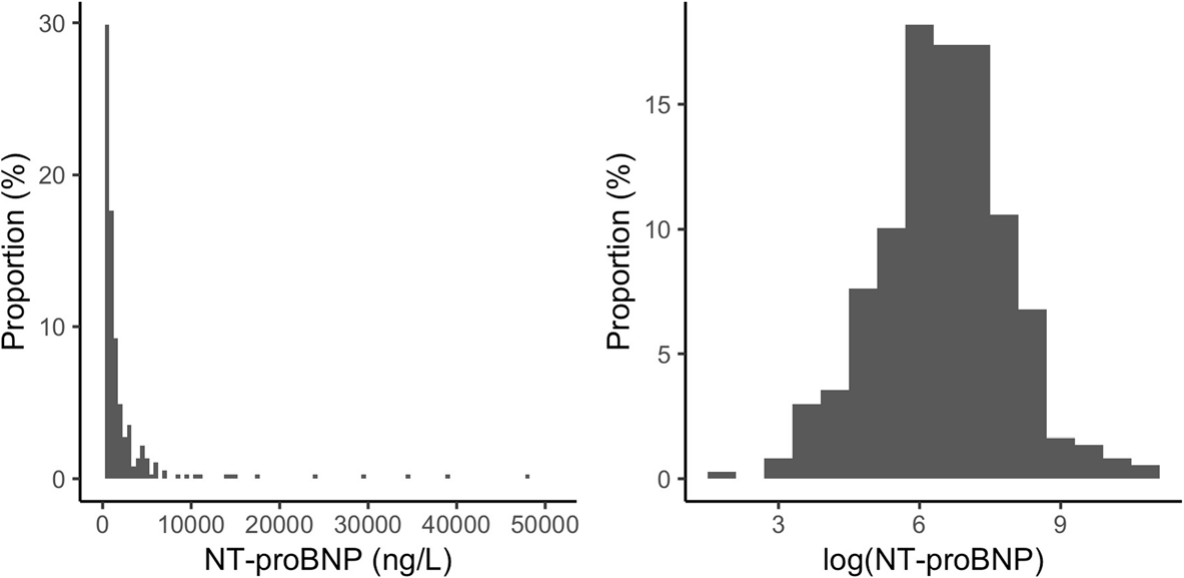
Distribution of NT-proBNP measurements at baseline with and without log transformation. Baseline serum N-terminal pro-B-type natriuretic peptide (NT-proBNP) levels were extremely right skewed. Log transformation resulted in a nearly normal distribution

In order to distinguish true progression of NT-proBNP from intra-individual variability, we applied the signal-to-noise ratio method with a random-effects linear regression model [20–22]. The model was previously demonstrated of good performance that was similar with a direct method (without modeling) of estimation, in studies investigating monitoring intervals of lipids and glycated hemoglobin [20–22]. Log transformation of NT-proBNP was used for model fitting, since in the study population the distribution of NT-proBNP had significant right skewness (Fig. 1) that was consistent with a previous report [23]. The response of the model (*Y*_*it*_ in Table 1) was {log (NT-proBNP) – log (baseline NT-proBNP)}. The model with random slopes and random intercepts assumed that each patient could have a different rate of increase over time. As covariates we included age, gender, year of the index admission, body mass index, smoking status, estimated glomerular filtration rate (eGFR) upon the admission, left ventricular ejection fraction measured on echocardiography during the admission, history of admission due to heart failure before the index admission, and comorbidity of hypertension, diabetes, dyslipidemia, atrial fibrillation, ischemic heart disease, and valvular disease, based on clinical significance [24–26].

**Table 1:** Equations used for the applied random-effects model

Equations or components	Interpretation
***Y***_***it***_***= β***_***i***_***× t + α***_***i***_***+ ε***_***it***_	Random-effects model predicting the observed value.
***Y***_***it***_	The value of {log (NT-proBNP) – log (baseline NT-proBNP)} of individual *i* at time *t.*
***α***_***i***_	Intercept for individual *i* in the model. ${\alpha}_i\sim \mathrm{N}\left(\alpha, {\sigma}_{\alpha}^2\right)$
***β***_***i***_	Progression rate of *Y*_*it*_ over time ${\beta}_i\sim \mathrm{N}\left(\beta, {\sigma}_{\beta}^2\right)$
***ε***_***it***_	Residual for individual *i* at time *t* in the model, reflecting measurement error and biological variability. ${\varepsilon}_{it}\sim \mathrm{N}\left(0,{\sigma}_{\varepsilon}^2\right)$
*t*	Time in month since baseline

The notation N(x, y) refers to a normal distribution with mean x and variance y. From this model, noise reflecting the scale of intra-individual variability equals the variance of the residual (i.e. ${\sigma}_{\varepsilon}^2$), whereas signal reflecting the scale of between-individual variability equals the variance of the progression rate multiplied by time (i.e. ${\sigma}_{\beta \mathrm{t}}^2={\sigma}_{\beta}^2\times {t}^2$)

*NT-proBNP* N-terminal pro-B-type natriuretic peptide

From the model above, we calculated between-individual variability (defined as signal) caused by different rates of increase as the variance of the random slope over time multiplied by time, and intra-individual variability (defined as noise) as the variance of the random residual. Equations in detail are shown in Table 1. Based on previous reports, the minimal informative interval was defined as the time when signal exceeds noise [20–22]. We calculated 95% confidence intervals (CI) for the informative intervals through non-parametric bootstrapping (1000 resampling with replacement, 1000 times) [27].

#### Stratified analysis according to underlying risks

Stratified analysis was performed according to age, gender, body mass index, left ventricular ejection fraction, renal function, presence of a history of multiple admissions due to heart failure, and the AHEAD risk score (0–1, 2–3, and 4–5). The AHEAD risk score comprises atrial fibrillation, hemoglobin < 13.0 g/dL in male or < 12.0 g/dL in female, elderly (> 70 years), abnormal renal function (creatinine > 1.47 mg/dL), and diabetes mellitus, with one point for each item [28].

Each subgroup was fitted to the model described above in order to calculate the signal, noise, and minimal informative monitoring interval.

## Results

### Participants and baseline characteristics

**Fig. 2:**
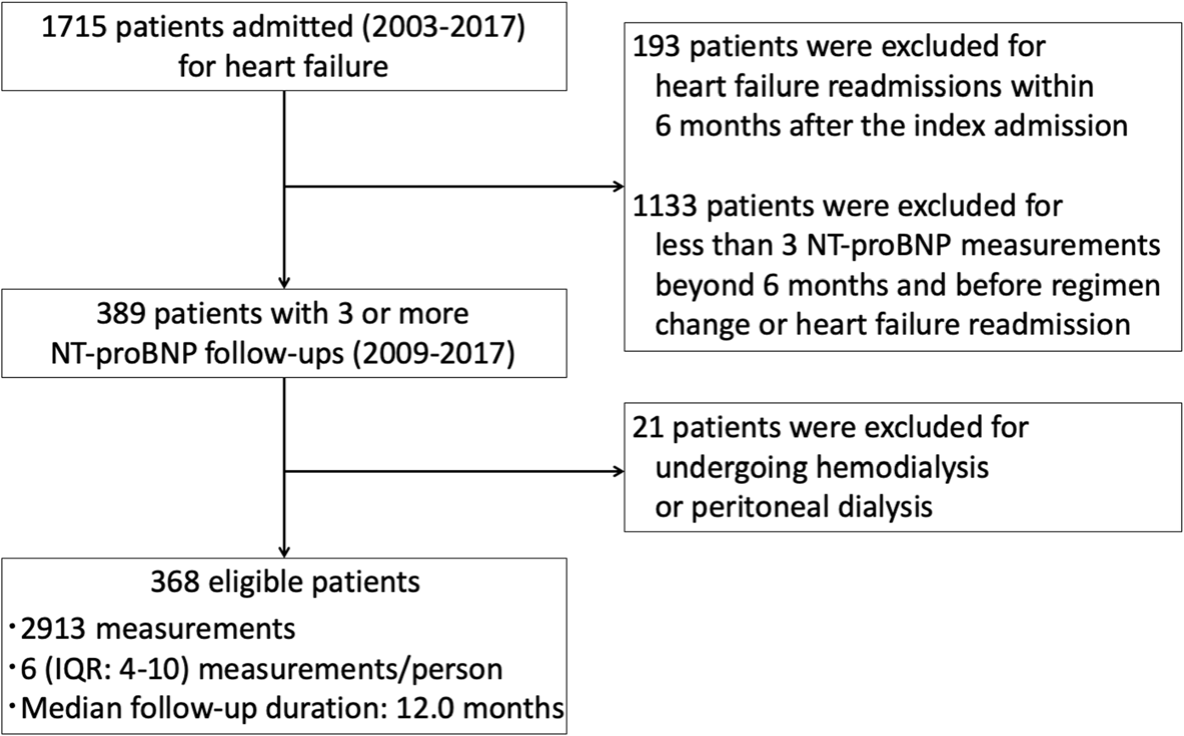
Scheme of inclusion and exclusion. *IQR* interquartile range, *NT-proBNP* N-terminal pro-B-type natriuretic peptide

Between January 2003 and December 2017, 1715 adult patients were admitted due to heart failure. Of those patients, 368 patients without end-stage renal disease had 3 or more NT-proBNP measurements during the analytic period (Fig. 2 and **Online Supplemental Fig.** **1**). All the serial NT-proBNP measurements were acquired between July 2009 and December 2017, since NT-proBNP measurement was available from July 2009 at the study site. The median analytic period was 12.0 months (IQR: 6.0–27.0). NT-proBNP was measured for a median 6 times (IQR 4.0–10.0) during the analytic period. The average of the individual mean measurement interval was 2.4 ± 2.4 months.

The median age of included patients was 75.5 years (IQR: 63.0–83.0), of whom 57% were male and 13% had a history of admission due to heart failure before the index admission. The median eGFR was 58.6 mL/min/1.73 m^2^ (IQR: 45.0–72.9), and the median left ventricular ejection fraction was 43.7% (IQR: 29.0–60.7). The patient baseline characteristics are summarized in Table 2 and the distributions of baseline serum NT-proBNP levels are illustrated in Fig. 1.

**Table 2:** Patient characteristics

Variables	Overall (*n* = 368)	
Age (y)	75.5	(63.0, 83.0)
Male gender	211	(57.3)
Year of admission		
2003 ~ 2007	28	(7.6)
2008 ~ 2012	147	(39.9)
2013 ~ 2017	193	(52.4)
Body mass index (kg/ *m*^2^)	23.3	(20.7, 26.6)
eGFR (mL/min/1.73 *m*^2^)	58.6	(45.0, 72.9)
Hemoglobin (g/dL)	12.6	(11.0, 14.3)
NT-proBNP on admission (ng/L)	2801.0	(1149.0, 5897.5)
LVEF on admission (%)	43.7	(29.0, 60.7)
Require respiratory support	86	(23.4)
Comorbidities		
Hypertension	277	(75.3)
Diabetes mellitus	109	(29.6)
Dyslipidemia	154	(41.8)
Atrial fibrillation	179	(48.6)
Ischemic heart disease	111	(30.2)
Valvular disease	219	(59.5)
Smoker	186	(50.5)
History of heart failure admissions before the index admission	49	(13.3)
Medications		
ACEI/ARBs	296	(80.4)
Beta-blockers	266	(72.3)
Mineralocorticoid receptor antagonists	186	(50.5)
Loop diuretics	247	(67.1)
Thiazides	22	(6.0)
AHEAD score		
0 ~ 1	124	(33.7)
2 ~ 3	215	(58.4)
4 ~ 5	29	(7.9)

Data are displayed as the median (interquartile range) for continuous variables and as a number (percentage) for categorical variables

*ACEI* Angiotensin converting enzyme inhibitor, *ARB* Angiotensin receptor blocker, *eGFR* Estimated glomerular filtration rate, *LVEF* Left ventricular ejection fraction, *NT-proBNP* N-terminal pro-B-type natriuretic peptide

### Signal, noise, and minimal informative intervals calculated from the random-effects model

**Fig. 3:**
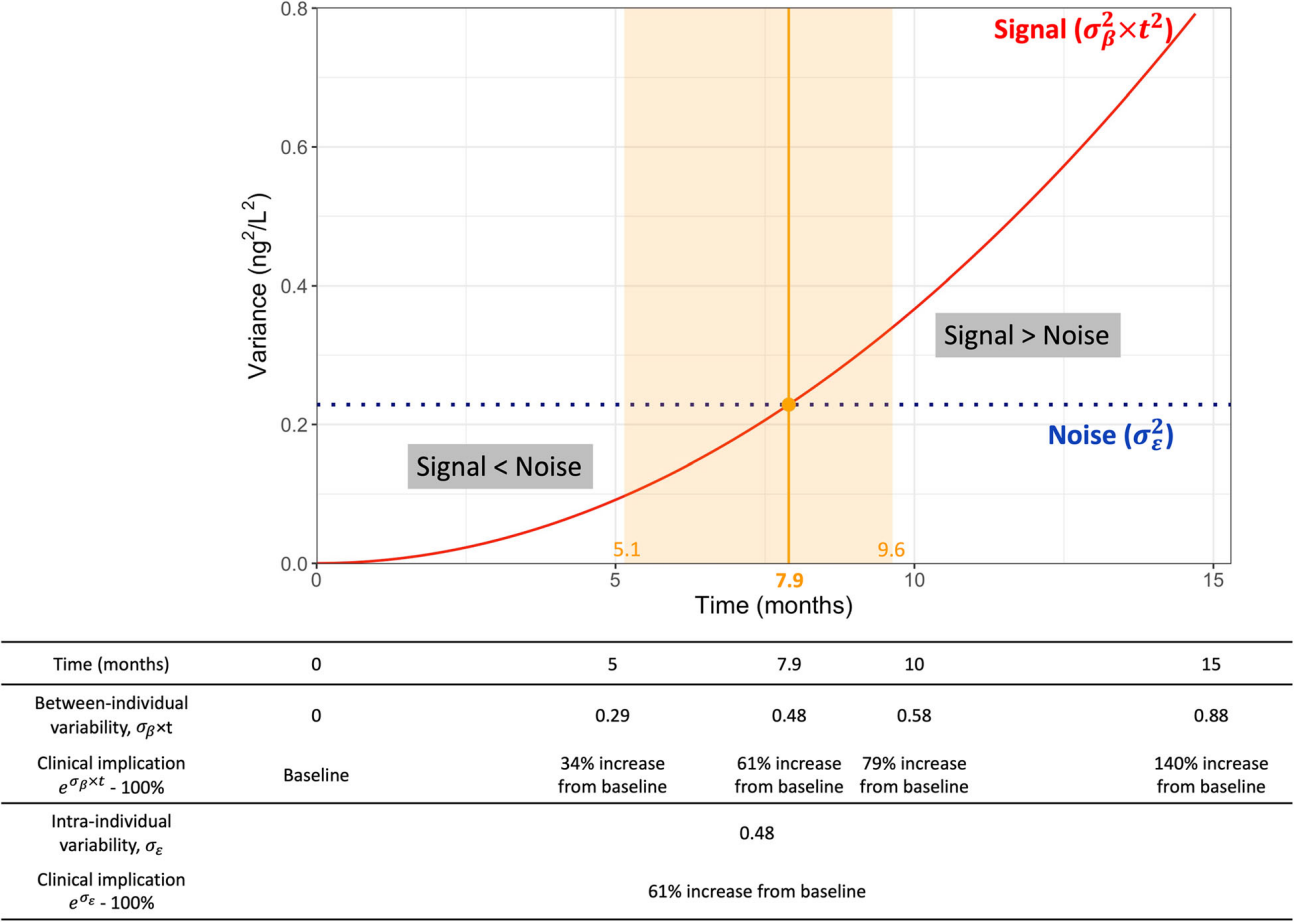
Curves of signal and noise in the overall stable heart failure population. Signal exceeded noise after 7.9 months from baseline. Noise corresponded to a 61% increase in serum N-terminal pro-B-type natriuretic peptide (NT-proBNP) from baseline

The fit of the random-effects model was substantial, with an intraclass correlation coefficient of 0.87 and a conditional r^2^ of 0.87 [29]. From the estimates of the random-effects model, we obtained the curves of signal and noise of NT-proBNP measurements (Fig. 3 and **Online Supplemental Table** **1**). The minimal informative interval, at which point signal exceeds noise, was 7.9 months (95%CI: 5.1–9.6) in the current population. The standard deviation of *ε*_*it*_ was 0.48, whose natural exponent was 1.61 (i.e. 161%), corresponding to a 61% increase of NT-proBNP from baseline.

**Fig. 4:**
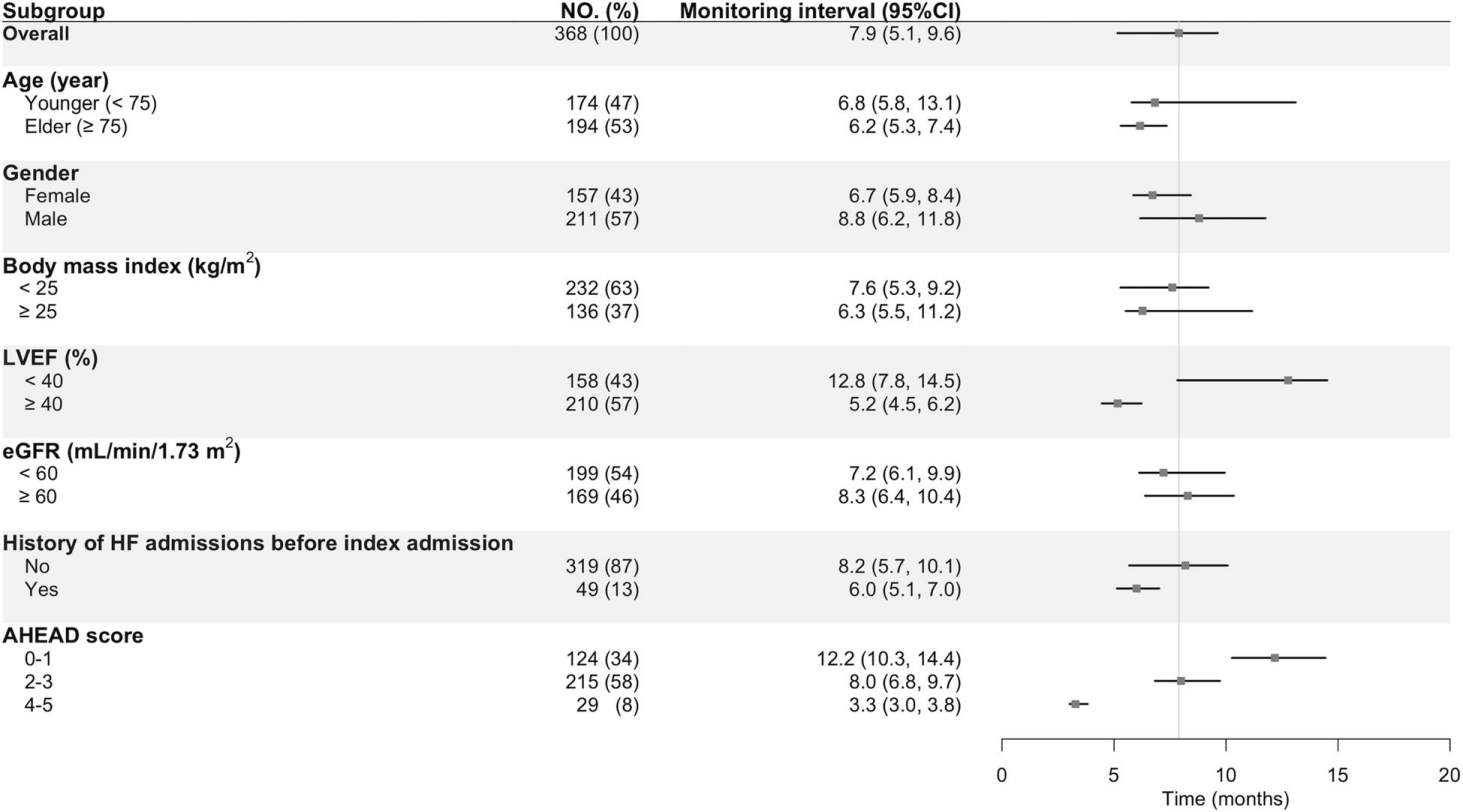
Minimal informative intervals of monitoring NT-proBNP in stable heart failure patients stratified by characteristics and risk. Patients with a preserved ejection fraction (left ventricular ejection fraction [LVEF] ≥ 40%) have a shorter informative interval than those with a reduced ejection fraction (LVEF < 40%). The informative interval decreases markedly as the AHEAD score increases. However, informative intervals do not differ significantly when stratified by age, gender, body mass index, and history of admissions due to heart failure before the index admission. *eGFR* estimated glomerular filtration rate, *HF* heart failure, *CI* confidence interval

Fig. 4 shows the minimal informative intervals for each stratification by age, gender, body mass index, left ventricular ejection fraction, renal function, history of heart failure admission before the index admission, and AHEAD score. Notably, as the AHEAD risk score increased, the minimal informative interval shortened significantly from 12.2 months (95%CI: 10.3–14.4) in those with scores of 0 or 1, to 3.3 months (95%CI: 3.0–3.8) in those with scores of 4 or 5. The patients with a left ventricular ejection fraction ≥40% had a shorter minimal informative interval than the patients with a left ventricular ejection fraction < 40% (5.2 months [95%CI: 4.5–6.2] vs 12.8 months [95%CI: 7.8–14.5]).

## Discussion

In the present study analyzing the retrospective data of patients with stable chronic heart failure, a minimal informative interval for monitoring serum NT-proBNP was 7.9 months (95%CI: 5.1–9.6) with a noise corresponding to a 61% change from baseline. More importantly, the minimal informative monitoring interval varied in accordance with different underlying risks. To the best of our knowledge, this is the first attempt to provide an evidence suggesting the informative monitoring interval of NT-proBNP in patients with stable chronic heart failure.

### Minimal informative monitoring interval of NT-proBNP taking into account intra-individual variability of NT-proBNP in stable chronic heart failure

NT-proBNP secretion levels are dynamic and depend on the different workloads [13]. Some small physiological stimuli, such as changes in sodium intake or physical exercise before blood test, may substantially influence serum NT-proBNP concentration measured in the outpatient clinic, resulting in biological variability [13]. This, along with measurement error, contributes to large-scale intra-individual variability, suggesting that frequent measurements are more susceptible to noise than to signal [13–16]. The present study indicates that the intra-individual fluctuation corresponds to a 61% change in the serum NT-proBNP level from baseline. This highlights the limited reliability of a single point measurement of NT-proBNP in the outpatient clinic, emphasizing the importance of other findings derived from symptoms, physical examination, or other tests such as echocardiography. Based on these results, a physician could be recommended in outpatient visits to place NT-proBNP orders after assessing other findings of the patient, and to avoid frequent NT-proBNP tests. While the minimal informative interval of 7.9 months from this study, an optimal monitoring interval of NT-proBNP measurement should be individualized according to the underlying risk profile, and be modified taking the test availability into account. Over-testing might lead to not only therapeutic confusion which might result in over-reaction to noise but also a huge amount of unnecessary costs, especially in healthcare contexts which allow liberal reimbursement for testing. Further studies investigating the financial impact of the minimal informative monitoring interval of NT-proBNP are warranted.

### Monitoring interval of NT-proBNP in NT-proBNP-guided therapy

Based on the results of the present study, increasing the length of the monitoring interval of NT-proBNP should be considered in heart failure patients with a stable status without changes in medication. However, in the midst of titration, the optimal monitoring interval of NT-proBNP is more debatable, especially in the case of NT-proBNP-guided therapy. NT-proBNP used as guidance might need to be monitored with a shorter interval due to a likely larger signal-to-noise ratio caused by intensification of treatment. The results of the present study could not be directly applied in such situation. In the Trial of Intensified vs Standard Medical Therapy in Elderly Patients with Congestive Heart Failure (TIME-CHF), Can Pro-brain-natriuretic Peptide Guided Therapy of Chronic Heart Failure Improve Heart Failure Morbidity and Mortality (PRIMA), and Guiding Evidence-based Therapy Using Biomarker-intensified Treatment in Heart Failure (GUIDE-IT) studies that failed to demonstrate prognostic superiority of NT-proBNP-guided therapy over conventional strategies in heart failure patients, patients were followed up every 3 months for NT-proBNP measurement [9, 10, 12]. The optimal monitoring interval during titration of treatment in NT-proBNP-guided therapy should be further investigated.

### Stratified analysis for minimal informative monitoring interval of NT-proBNP

The minimal informative monitoring interval shortened along with the accumulation of risk represented by an increase of the AHEAD score. The present results indicate the need for tailored monitoring intervals for NT-proBNP levels in stable heart failure patients based on underlying risks. The difference in minimal informative intervals between patients with higher and lower ejection fractions was driven by a significantly smaller noise in the higher ejection fraction subgroup (1.89 ×10^−1^ ng^2^/L^2^ [95%CI: 1.65 ×10^−1^ to 2.03 ×10^−1^]) than in the lower ejection fraction subgroup (2.61 ×10^−1^ ng^2^/L^2^ [95%CI: 2.36 ×10^−1^ to 2.93 ×10^−1^]). This was inconsistent with the results from 2 previous studies, which suggested that variability in NT-proBNP was not influenced by the left ventricular ejection fraction [30, 31]. However, one of those studies only recruited subjects with an ejection fraction > 50% [31]. while the other mostly included patients with an ejection fraction < 40% [30]. The present study included both evenly and for the first time demonstrated that the intra-individual variability was smaller in patients with a preserved ejection fraction, compared with those with a reduced ejection fraction.

### Study limitations

This study has several main limitations. First, this is a single center retrospective cohort study with a limited number of patients. Decisions regarding the schedule of NT-proBNP measurement, as well as alteration of medication regimen or readmission were left to the physicians’ discretion. The results of the present study should be externally validated. Second, the patients readmitted for heart failure within 6 months after the index admission, who might have benefitted the most from NT-proBNP measurement, were excluded. Finally, our model was based on the assumption that log (NT-proBNP) changes linearly in patients with a stable status of chronic heart failure. The linearity of its change remains to be further investigated.

## Conclusions

Utilizing a statistical method taking into consideration the biological variability and measurement error of the NT-proBNP level, we determined that the overall minimal informative interval for monitoring NT-proBNP levels was 7.9 months (95%CI: 5.1–9.6) in the current population, while the intra-individual variability corresponded to a 61% increase from baseline. Of note, the minimal informative monitoring interval was shorter in patients with a worse predicted prognosis. These results emphasize the limitation of frequent measurement of NT-proBNP, the importance of detecting signs of heart failure exacerbation other than NT-proBNP, as well as the necessity of individualization in planning follow-up visits according to different risk profiles.

## Supplementary information

**Additional file 1: Supplemental Table 1**. Estimates of the random-effects model for the overall stable heart failure population. **Supplemental Fig. 1**. Schematic of analytic period and follow-up NT-proBNP measurements.

## Data Availability

The datasets generated and analyzed in the current study are not publicly available due to restrictions by the institutional research board.

## Abbreviations

NT-proBNP: N-terminal pro-B-type natriuretic peptide
IQR: Interquartile range
SD: Standard deviation
eGFR: Estimated glomerular filtration rate
CI: Confidence interval
