# Supplementary material for: The minimal informative monitoring interval of N-terminal pro-B-type natriuretic peptide in patients with stable heart failure
Source: BMC Cardiovasc Disord. 2020 Jun 1;20:262. doi: 10.1186/s12872-020-01537-7 (PMC7268659; doi:10.1186/s12872-020-01537-7)
Supplement: Supplementary file 1 — Additional file 1: Supplemental Table 1. Estimates of the random-effects model for the overall stable heart failure population. Supplemental Fig. 1. Schematic of analytic period and follow-up NT-proBNP measurements. [file 12872_2020_1537_MOESM1_ESM.docx]

**Additional File 1 to:**

**The minimal informative monitoring interval of N-terminal pro-B-type natriuretic peptide in patients with stable heart failure**

Zhehao Dai, Taku Asano, Osamu Takahashi, Nobuyuki Komiyama, Sachiko Ohde

**Supplemental Table 1. Estimates of the random-effects model for the overall stable heart failure population**

| Estimates of fixed effects | |
| --- | --- |
| Fixed effect of *α* | -3.72 |
| Fixed effect of *β* | $1.58 \times{10}^{-2}$ |
| Estimates of random effects | |
| $\sigma_{\alpha}^{2}$ | $4.29 \times{10}^{-2}$ |
| $\sigma_{\beta}^{2}$ | $3.67 \times{10}^{-3}$ |
| $\sigma_{\varepsilon}^{2}$ | $2.29 \times{10}^{-1}$ |

**Supplemental Fig. 1. Schematic of analytic period and follow-up NT-proBNP measurements**


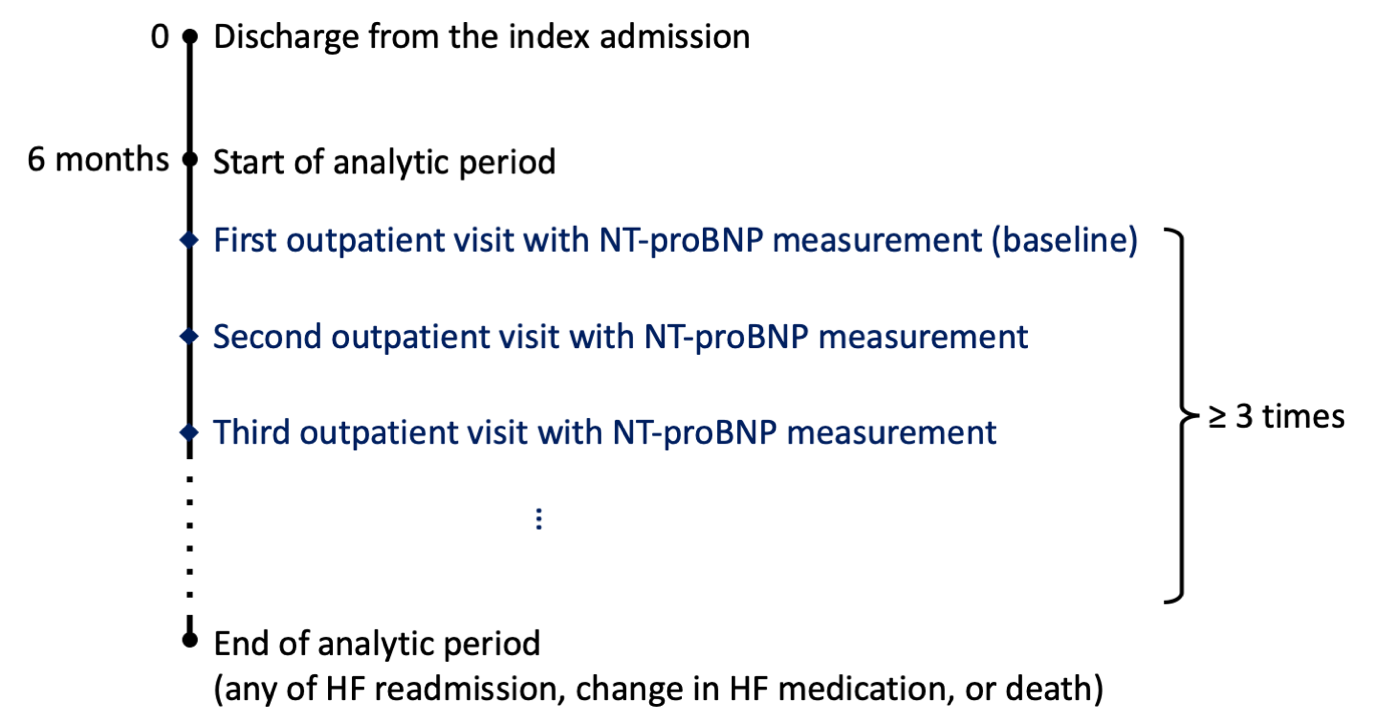


*HF* heart failure, *NT-proBNP* N-terminal pro-B-type natriuretic peptide.
